# Aortic Deceleration Injury Treated by Endograft: A Case Report with 11-Year Followup

**DOI:** 10.1155/2012/608358

**Published:** 2012-12-24

**Authors:** Zhi-Wei Hu, Wang-Zhong Gao, Yong-Quan Gu, Bing Chen, Guang-Chang Zhu, Wei-Tao Liang, Ya-Chan Ning

**Affiliations:** Vascular Surgery, Xuanwu Hospital, Capital Medical University, No. 45 Changchun Street, Xicheng District, Beijing 100053, China

## Abstract

Aortic deceleration injury is a common and critical condition following automobile accident with high fatality. The survivors complicated with associated serious injuries are even rare and definitive treatment is required. A 37-year-old male patient had both aortic blunt injury and coronary artery injury after a frontal car collision. After failed coronary artery percutaneous transluminal angioplasty (PTA) and deteriorated aortic lesion, the ruptured aorta was subsequently successfully treated by us with a self-made individualized endograft. The endograft was well in position and the patient functioned well in 11-year followup. With the development of endograft and technique, the endovascular treatment may be an option for patients with complicated aortic blunt injury. Yet careful patient selection and the long-term followup are essential.

## 1. Background

Aortic deceleration injury is a common and critical condition following automobile accident with high fatality. The survivor of aortic combined with cardiac injury, even coronary artery injury, are fewer. For the survivors of the initial injury, a majority will die without definitive treatment. Major operations are mandatory for some of the patients. However, the patients complicated with associated serious injuries. Cardiac injury, delaying aortic repair, was suggested, as the emergence of endovascular treatment with minimal invasive advantage, and endograft may be an option for this group of patients. Here is such a patient treated with endovascular treatment with 11 years followup.

## 2. Patient

On April 16, 2001, a 37-year-old driver was admitted to the Department of Cardiology from emergency service. He had a head-on care collision a week earlier without treatment, which caused him chest tightening, pain, breathlessness, and a transient unconsciousness. His vital sign was stable (pulse rate 80 beat/min, respiratory rate 20 times/min, blood pressure 140/70 mmHg) and no signs of visible injury were found. But a CT scan revealed a widening mediastinum and a massive left pleural effusion. He had undertaken medical treatment and monitoring.

On the seventh day of admission, he complained a severe chest pain. Electrocardiogram showed a ventricular fibrillation. A defibrillation was successful, but the ST segment remained elevated on the lead V1–V5, with cardiac enzyme: CK3 974 U/L and CK-MB 469 U/L. Echocardiography showed no contraction of the anterior cardiac wall and ejection fraction (EF) 36%, indicating a myocardial infarction. Coronary angiography revealed a disruption of the proximal left anterior descending artery with a 95% stenosis. A PTA was unsuccessful.

**Figure 1:**
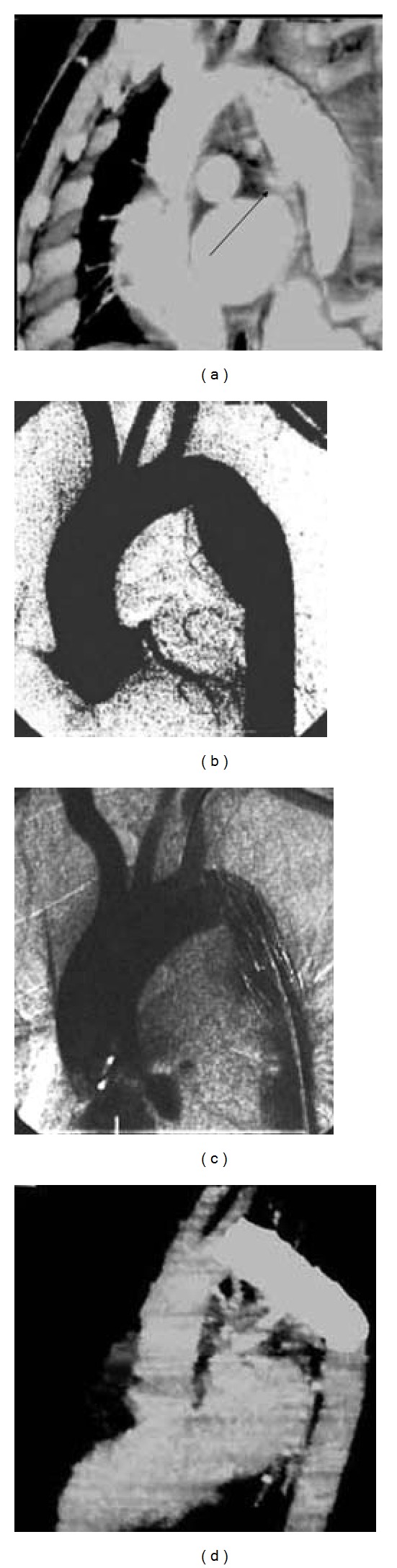
(a) MRI showing a spindle-shaped aneurysm at the beginning of the descending aorta with a suspected rupture in its inner wall (arrow); (b) DSA showing a fusiform aneurysm at the top of the descending aorta; (c) the lesion is properly treated by an endograft; (d) follow-up MRI showing a well-deployed endograft two years after deployment.

Twenty days later, the patient was transferred to our department, and MRI revealed a spindle-shaped aneurysm at the aortic isthmus with an irregular inner contour. A rupture of interior wall was suspected (Figure 1(a)). On May 19, 2001, from right femoral approach, a 32 mm × 120 mm individualized indigenous endograft (stainless steel stent with silk membrane) was made and successfully deployed under fluoroscopic observation. Angiography showed a successful treatment (Figures 1(b) and 1(c)). The patient was discharged in a satisfactory condition seven days later, with EF improved to 47.9%. Follow-up MRI was obtained two years later (Figure 1(d)).

**Figure 2:**
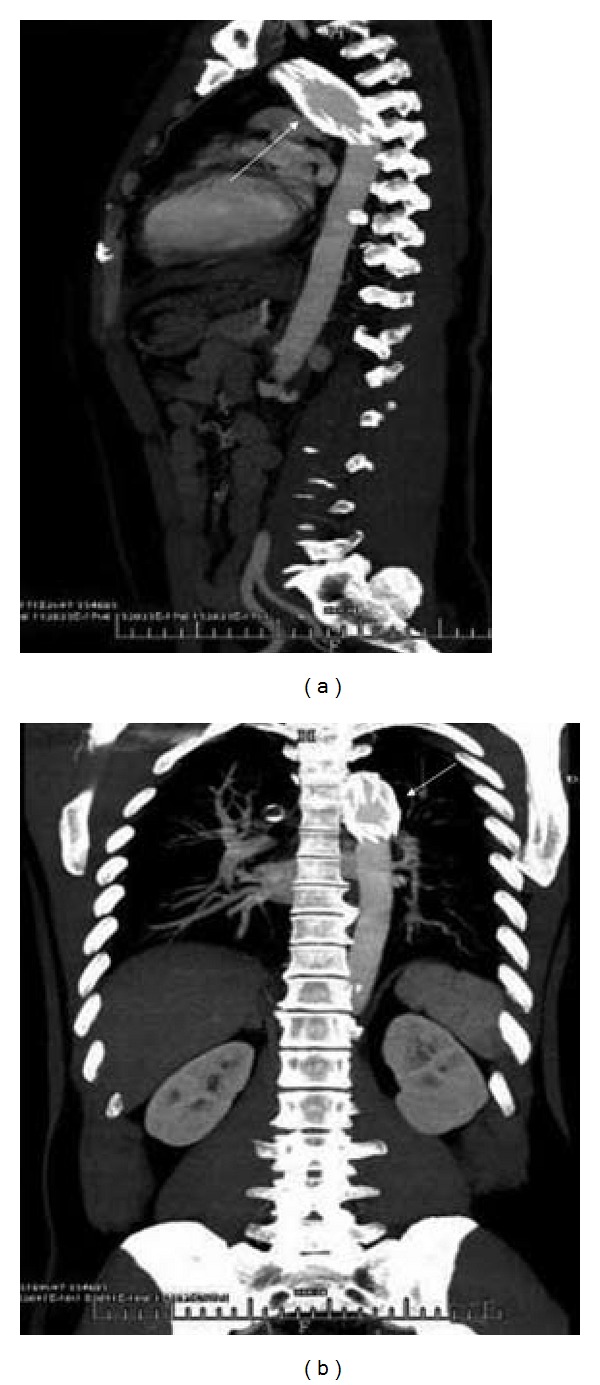
The CTA 10 years after endovascular treatment: the endograft remained in the original position with slight dilation at its center (arrow).

In October 2010, the patient came back for checkup, informing that he had resumed working shortly after discharge, not as a driver, but as a security guard. He enjoyed such a good health that it took him only six minutes to run 1000 meters. Even though his echocardiography still showed no movement of the anterior cardiac wall, he was in good shape. A computed tomographic angiography (CTA) followup confirmed that the endograft was in the same position vis-a-vis the isthmus. Although the middle portion of the graft was slightly dilated, it was acceptable at this point (Figure 2). In July 2012, eleven years after endograft deployment, he remained healthy, with all functions normal. 

## 3. Discussion

As high as 80% traumatic injury of the aorta patients die at the scene of the accident [1]. Acute rupture of the thoracic aorta is associated with other serious injuries attributable to the action of violent forces of acceleration or deceleration. More than half of those with aortic blunt rupture have concomitant heart and pericardium injuries. Heart and pericardium ruptures are most common among fatally wounded car drivers. In frontal car collision fatalities, the right atrium is the most vulnerable and frequently injured part [2]. Therefor there are very few survivors of aortic blunt injury complicated with heart, and most of them would die without definitive treatment. The accompanying coronary artery injury should be part of heart injury, but it is seldom reported [3]. In addition to aortic isthmus and heart injury, the associated injuries can account for as high as 90% of mortality in patients with traumatic aorta injury [4], which may render such patients unsuitable for immediate open surgical repair. Strict controls on blood pressure and heart rate to decrease the shear force on the aortic wall and close radiographic followup are all necessary. A delayed approach for patients with substantial injuries was considered to be rational, safe and allows management of coexisting life-threatening conditions [5, 6]. However, a portion of patients still died of in-hospital aortic rupture [7], and the critical condition was about to occur in this particular case while it was 50 days after the injury.

In a perfect storm, this patient also had coronary injury and myocardial infarction. A major surgery would be more risky at that time. At the time in 2001, we still did not have endovascular device available in China, the one that we used was already our 41th indigenous endograft since 1995. The stent was manually constructed in Z shape and the covering was a piece of a properly sized Chinese silk membrane. They were assembled together by propylene suture [8, 9]. The plastic sheath used in this case was F 24. In a few cases after this case, this kind of endograft has been formally manufactured by the Yu Hen Jia Technology Company, Beijing, which was approved by CDA (Chinese Food and Drug Administration) and later on it even developed a subclavian branched endograft with advantage of stabilization of the main graft during the treatment of Stanford B dissection [10]. 

The endograft in the early days that we tailored for him was successfully deployed, thus avoided a major surgical repair. Followed up for 11 years, the endograft was stable, with just a slight dilatation at its middle part, which is well within the range of tolerance. Endografting is a good option, as it essentially adds no physiological burden; no thoracotomy, systemic anticoagulation, aortic clamping, or declamping is required; and hospital stay is shorter. Prognosis for selective endovascular repair of blunt thoracic aortic injury is comparable to that for open surgery. The development of endograft repair refined our abilities to even severely injured or frail patients with substantially reduced procedure-related rates of death and paraplegia. However, there is a risk of reintervention, especially in young patients [11, 12]. Nevertheless endografts need further improvement both in material and in design and even the commercial available armament has been evolved for decades. Last but not least, a careful selection of patients and a long-term followup are both essential, especially in young patients.
